# Prognostic factors for mortality in invasive pneumococcal disease in adult: a system review and meta-analysis

**DOI:** 10.1038/s41598-021-91234-y

**Published:** 2021-06-04

**Authors:** Hao Chen, Hiromi Matsumoto, Nobuyuki Horita, Yu Hara, Nobuaki Kobayashi, Takeshi Kaneko

**Affiliations:** Department of Pulmonology, Yokohama City University Graduate School of Medicine, 3-9 Fukuura, Kanazawa, Yokohama, 236-0004 Japan

**Keywords:** Diseases, Infectious diseases, Bacterial infection

## Abstract

Risk factors associated with mortality in invasive pneumococcal disease remain unclear. The present work is a meta-analysis of studies that enrolled only patients with invasive pneumococcal disease and reported on mortality. Potentially eligible reports were identified from PubMed, CHAHL, and Web of Science, comprising 26 reports in total. Overall mortality for invasive pneumococcal disease was reported as 20.8% (95% confidence interval (CI) 17.5–24%). Factors associated with mortality were age (odds ratio (OR) 3.04, 95% CI 2.5–3.68), nursing home (OR 1.62, 95% CI 1.13–2.32), nosocomial infection (OR 2.10, 95% CI 1.52–2.89), septic shock (OR 13.35, 95% CI 4.54–39.31), underlying chronic diseases (OR 2.34, 95% CI 1.78–3.09), solid organ tumor (OR 5.34, 95% CI 2.07–13.74), immunosuppressed status (OR 1.67, 95% CI 1.31–2.14), and alcohol abuse (OR 3.14, 95% CI 2.13–4.64). Mortality rates with invasive pneumococcal disease remained high, and these findings may help clinicians provide appropriate initial treatment for this disease.

## Introduction

*Streptococcus pneumoniae* commonly colonizes the upper airway asymptomatically, and is the cause of approximately 25–50% of cases of community-acquired pneumonia and meningitis^1^. The most severe form of pneumococcal infection, invasive pneumococcal disease (IPD), includes bacteremic pneumonia, meningitis, and septicemia with significant morbidity and mortality. In the United States of America, data from the Centers for Disease Control and Prevention showed 9.6 cases of IPD per 1,00,000 persons in all age groups^2^. In England and Wales, an IPD incidence of 7 per 1,00,000 persons in all age groups was reported, increasing to 21 per 1,00,000 among persons ≥ 65 years old^3^. The case fatality rate of IPD may reach 15–20% in adults and 30–40% in the elderly^4^.

A marked reduction in the rate of IPD due to pneumococcal conjugate vaccine and protein conjugate vaccine 13 serotypes has been reported for individuals ≥ 55 years old, with a much smaller decline in those 17–54 years old^5^. In the prospective international observational study, the overall mortality rate of pneumococcal bacteremia was 17%^6^. Although various articles have discussed risk factors for mortality from IPD, an overview has been lacking. This study was therefore designed to clarify prognostic factors contributing to death due to IPD.

## Methods

### Overview

The study protocol followed the Meta-analysis Of Observational Studies in Epidemiology (MOOSE) group statement and was registered to the University Hospital Medical Information Network (ID: UMIN000041377)^7, 8^. The need for institutional review board approval was waived because of the systematic review nature of this research. H.C. and M.M. independently performed the searching process and data extraction, and subsequently built consensus results.

### Search of the literature

Three major databases (Medline, CHAHL, and Web of Science) were searched on July 30, 2020. The two reviewers independently extracted and recorded data for a predefined checklist including the following items: study characteristics (i.e., country and year of study), characteristics of the cohort, and case definitions. The following search formula was used: (“invasive pneumococcal disease” OR “pneumococcal infection” OR “*Streptococcus pneumoniae* infection” OR “pneumococcal bacteremia”) AND (“mortality” OR “death”) AND (“odds ratio” OR “relative risk” OR “hazard ratio”).

### Inclusion and exclusion criteria

No restrictions were placed on article types or publication language. To be included, a study had to include: 1) patients with IPD; 2) data outlining risk factors for 30-day mortality or in-hospital mortality after multivariable regression analysis; and 3) no restrictions on vaccination.

Exclusion criteria were as follows: (1) patients < 15 or > 65 years old only; (2) risk factors assessed by univariable analysis only; (3) risk factors assessed in categories of subgroup only; or (4) patients with human immunodeficiency virus (HIV) infection only.

### Definitions

IPD was defined as an illness occurring in association with the isolation of *S. pneumoniae* from a normally sterile body specimen including blood, cerebrospinal fluid, peritoneal, pleural or synovial fluid, or abscess aspirates or tissue specimens/swabs obtained intraoperatively, but excluding bronchoalveolar lavage. Immunosuppressed status was defined as HIV infection, splenectomy, hematological malignancy, autoimmune disorder, presence of a transplant, or cancer chemotherapy within 4 weeks before the onset of bacteremia. Septic shock was defined as sepsis-induced hypotension, persisting despite adequate fluid resuscitation, along with the presence of hypoperfusion abnormalities or organ dysfunction^9^.

### Outcomes

Potential prognostic factors were defined in this study as any clinical information related to mortality such as age, sex, underlying diseases, focus of infection, and place of infection acquisition. Risk factors for 30-day and in-hospital mortalities were analyzed separately if > 3 articles were found in each subgroup.

### Quality assessment

Two reviewers independently assessed the methodological quality of selected studies using the Newcastle–Ottawa Scale quality assessment to evaluate the quality of observational studies. Disagreements among reviewers were discussed, with agreement reached by consensus^10^.

### Statistics

All analyses were performed using Review Manager version 5.3 (Cochrane Collaboration, Oxford, UK). Figures illustrated using Review Manager were adjusted as necessary. Prognostic factors for 30-day and in-hospital mortality rates were merged because of their similarity after meta-analysis. Heterogeneity evaluated using I^2^ statistics was interpreted as follows: I^2^ = 0%, no heterogeneity; I^2^ > 0% but < 25%, minimal heterogeneity; I^2^ ≥ 25% but < 50%, mild heterogeneity; I^2^ ≥ 50% but < 75%, moderate heterogeneity; and I^2^ ≥ 75%, strong heterogeneity^11^.

## Results

### Study search

**Figure 1:**
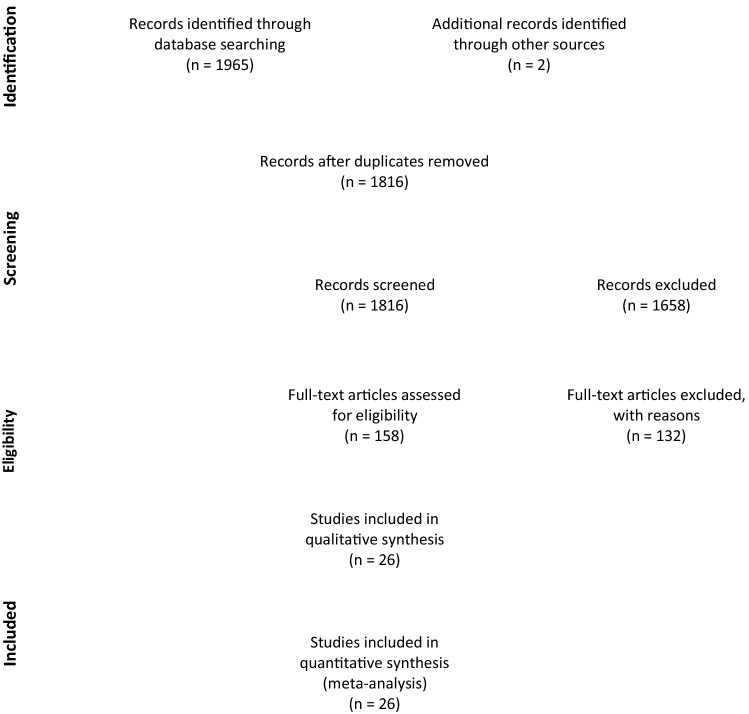
PRISMA flow chart for study selection.

Of these 26 articles identified, 2 demonstrated data from two independent populations (Fig. 1). Our analysis thus eventually extracted data from 16 countries for 27,742 patients with IPD, of whom 5810 died.

### Characteristics of included studies

The 26 articles were published between 2000 and 2020, reported from Spain and the United States (n = 3 each), Canada, Japan, Israel, South Korea, the Netherlands, the United Kingdom, and Taiwan (n = 2 each), and 6 other countries (n = 1 each)^6, 12–36^. Two articles from Sweden and Georgia were multi-country studies compromising patients from 5 and 10 countries (Table 1). Twenty-one studies assessed either only adults or only adults plus adolescents ≥ 15 years old. Six studies included patients with IPD at any age. Six studies included only patients with HIV infection. Only two studies discussed status of HIV infection separately and the remaining four studies classified HIV as one condition defining an immunosuppressed status. Eight studies did not discuss the status of pneumococcal conjugate vaccination, seven studies revealed missing data for vaccination status, and nine studies discussed vaccination status (vaccination rates, 0–54.5%). No study identified a relationship between vaccination and mortality in multivariable analysis. Thirteen studies discussed in-hospital mortality as an outcome, twelve studies discussed 30-day mortality rates, and one study discussed 28-day mortality rate. The median in-hospital mortality was 23.0% (95% confidence interval (CI) 17.2–27.2%) and the median 30-day mortality rate was 18.9% (95% CI 13.9–23.9%). The overall mortality rate from IPD included in this study was 20.8% (95% CI 17.5–24.0%). Median Newcastle–Ottawa Scale score was 6, suggesting that most studies were of acceptable quality.

**Table 1:** Background characteristics of the 26 studies included.

Authors	Year	Country	N	Mortality	Risk factors in summary	NOS
Farinas-Alvarez	2000	Spain	156	33.9%^a^	Age, severity of illness, development of complications, parenteral nutrition	7
Kalin	2000	Sweden^d^	460	11.5%^a^	Age > 65 years, nursing home, chronic lung disease, acute physiology score	7
Fernndez-Guerrero	2003	Spain	156	33.9%^b^	Multilobe pneumonia, inappropriate therapy, obtundation, nosocomial infection	6
Yu	2003	USA	844	16.9%^a^	Age > 65 years, critical illness, immunodeficiency, nosocomial infection, underlying chronic disease, organism susceptibility	8
Maugein	2003	France	436	20.6%^a^	Age > 60 years, immunodeficiency, focus of infection, nosocomial infection	7
Neuman	2007	USA	1574	13.0%^a^	Age, male, race, nosocomial infection, immunodeficiency, focus of infection, organism susceptibility, others	7
Alanee	2007	Georgia^e^	796	19.0%^a^	Age ≥ 65 years, serotype, focus of infection, immunosuppression, chronic lung disease, suppurative lung complications	8
Lin	2010	Taiwan	302	19.2%^b^	Age ≥ 65 years, nosocomial infection, immunosuppression, solid organ tumor, liver disease, heart disease	7
Lujan	2010	Spain	299	11.0%^b^	Age, serotype, CCI, PSI	7
Mooiweer	2011	Netherlands	204	16.0%^a^	Age appropriate treatment, CRP level	7
Song	2012	Korea	150	26.7%^b^	Age ≥ 65 years, solid organ tumor, focus of infection, neutropenia, APACHE II score	7
Kang	2013	Korea	136	26.5%^b^	Immunosuppression, septic shock, development of ARDS, levofloxacin resistance	8
Suzuki	2013	Japan	135	25.0%^a^	Age, sex, CCI, septic shock, antibiotic selection	7
Rudnick	2013	Canada	6404	16.4%^a^	Age, nursing home, immunosuppression, underlying chronic disease, alcohol abuse, focus of infection, serotype	7
Regev-Yochay	2013	Israel	460	18.0%^a^	Age, metastatic malignancy, focus of infection, chronic renal disease	7
Cohen	2015	South Africa	3953	34.0%^a^	Age ≥ 65 years, focus of infection, serotype, HIV, province poverty level	6
Hanada	2016	Japan	505	24.1%^c^	Age, underlying disease, mechanical ventilation, serotype	7
Hughes	2016	UK	1316	18.5%^b^	Age, sex, focus of infection, underlying disease, serotype	7
Wagenvoort	2016	Netherlands	960	7.0%^b^	Age ≥ 65 years, immunosuppression, chronic kidney disease, chronic lung disease, chronic cardiovascular disease	7
Askim	2016	Norwegian	414	12.3%^b^	Age, sex, CCI, comorbidities	7
Lee	2018	Taiwan	134	16.4%^b^	Serotype, CCI, Pitt bacteremia score, inappropriate treatment	7
Kim	2018	Korea	319	34.2%^a^	Age ≥ 65 years, focus of infection, Pitt bacteremia score	7
Regev-Yochay	2018	Israel	2345	30.2%^a^	Age ≥ 65 years, focus of infection, risk group, serotype	7
Lemay	2019	Canada	1719	24.9%^b^	Age, male, homeless, alcohol abuse, immunosuppression, focus of infection, serotype	7
Houseman	2019	UK	2510	19.0%^b^	Age, male, focus of infection, risk group, alcohol abuse, serotype	7
Man	2020	Hong Kong	792	11.5%^b^	Age > 65 years, chronic kidney disease, septic shock, positive urinary antigen test	7

^a^in-hospital mortality;

^b^30-day mortality;

^c^28-day mortality.

^d^United States of America, Canada, Spain, United Kingdom, Sweden.

^e^South Africa, United States of America, Sweden, Spain, New Zealand, Taiwan, Argentina, Brazil, Hong Kong, and France.

NOS: Newcastle–Ottawa Scale score; score ranges from 0 (worst) to 9 (best).

### Meta-analysis

**Figure 2:**
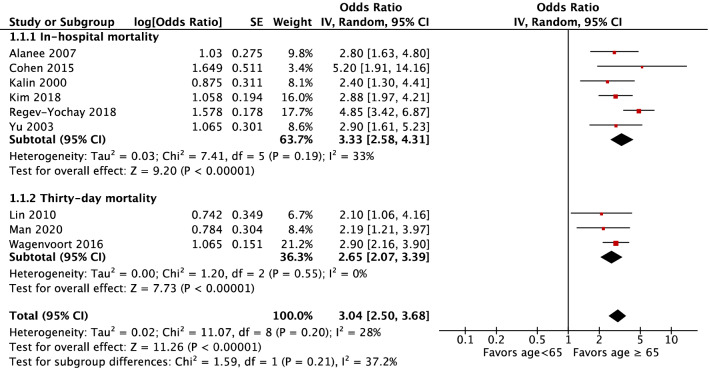
Risk factor of Age > 64 years.

According to a random-model meta-analysis, study participants > 64 years old were at higher risk of in-hospital or 30-day mortality compared to those < 65 years old in subgroups (odds ratio (OR) 3.33, 95% CI 2.58–4.31, *P* < 0.001 and OR 2.65, 95% CI 2.07–3.39, *P* < 0.001, respectively) (Fig. 2). The total effect of age > 64 years old was a high risk for overall mortality (OR 3.04, 95% CI 2.50–3.68, *P* < 0.001, I^2^ = 37.2%). The I^2^ statistic of 37.2% suggested mild heterogeneity of this model. No heterogeneity between in-hospital mortality and 30-day mortality comparisons was revealed (*P* = 0.21).

**Figure 3:**
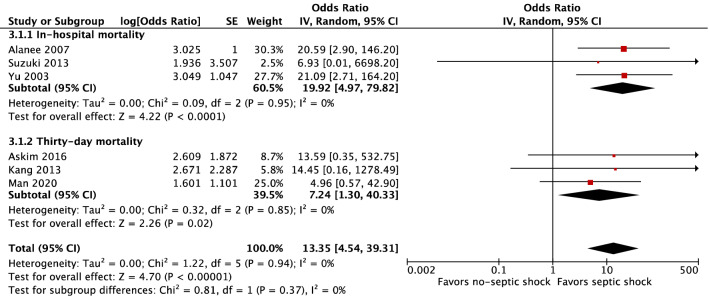
Risk factor of septic shock.

Patients in a state of septic shock on admission demonstrated higher risks of in-hospital and 30-day mortality (OR 19.92, 95% CI 4.97–79.82, *P* < 0.001 and OR 7.24, 95% CI 1.30–40.33, respectively, *P* = 0.02) (Fig. 3). The total effect of septic shock was a high risk for overall mortality (OR 13.35, 95% CI 4.54–39.31, *P* < 0.001, I^2^ = 0%). The I^2^ statistic of 0% suggested no heterogeneity for this model. No heterogeneity between in-hospital mortality and 30-day mortality comparisons was revealed (*P* = 0.37).

**Figure 4:**
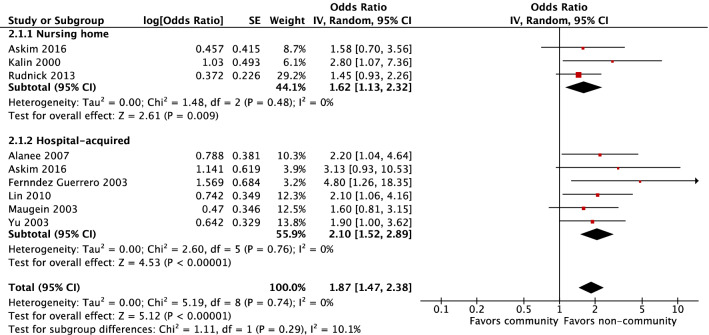
Risk factor of community-acquired infection versus.

Residence in a nursing home and nosocomial infection were associated with higher risks of mortality compared to community-acquired infection (OR 1.62, 95% CI 1.13–2.32, *P* = 0.009 and OR 2.10, 95% CI 1.52–2.89, *P* < 0.001, respectively) (Fig. 4).

Non-community-acquired infection showed a higher risk of mortality than community-acquired infection (OR 1.87, 95% CI 1.47–2.38, *P* < 0.001, I^2^ = 10.1%). The I^2^ statistic of 10.1% suggested minimal heterogeneity of this model.

Underlying chronic diseases also showed a risk of mortality (Figure S1). Liver disease, chronic kidney disease, and cardiovascular disease were frequently discussed (OR 2.73, 95% CI 1.57–4.76, *P* < 0.001; OR 2.99, 95% CI 2.09–4.27, *P* < 0.001; and OR 1.65, 95% CI 1.25–2.19, *P* < 0.001, respectively). The total effect of underlying chronic disease on mortality was 2.34 (95% CI 1.78–3.09, *P* < 0.001). The I^2^ statistic of 72.9% suggested strong heterogeneity for this model.

Immunosuppressed status was a prognostic factor for mortality from IPD (OR 1.51, 95% CI 1.24–1.84, *P* < 0.001, I^2^ = 47%) (Figure SS2. Only two studies discussed HIV infection separately (OR 4.28, 95% CI 1.98–9.24, *P* < 0.001, I^2^ = 0%). Four studies discussed solid organ tumor as a risk factor for mortality (OR 5.34, 95% CI 2.04–13.74, *P* = 0.003, I^2^ = 79%) (Figure S3. Alcohol abuse also increased mortality from IPD (OR 3.14, 95% CI 2.13–4.64, *P* < 0.001) (Figure S4).

## Discussion

Despite the wide adoption of pneumococcal conjugate vaccination, the overall mortality rate from IPD has remained high, at 20.8%. This meta-analysis revealed older age (> 64 years old), septic shock, immunosuppressed status, underlying chronic diseases, solid organ tumor, alcohol abuse, nursing home, and nosocomial infection were prognostic factors for mortality from IPD. These results appear useful in understanding prognostic factors for mortality due to IPD because of the solid methodology in accordance with the MOOSE statement, with statistical power supported by more than 27,000 subjects.

Aging is a major risk factor for the development of virtually every lung disease, increasing both morbidity and mortality, while morbidities and mortalities from other prevalent diseases have declined or remained stable^37^. The conventional nuclear family model is becoming increasingly uncommon, and the majority of elder care is provided by relatives, albeit with varying patterns of involvement and responsibility across family structures^38^. Residence in a nursing home and nosocomial infection have been associated with the progression of aging. Comorbidity also plays an important role in affecting mortality. This study identified underlying chronic diseases, solid organ tumor, and immunosuppressed status as important factors to clinical progress.

Septic shock is a frequent complication of pneumococcal infection and causes high rates of morbidity and mortality^39^. The presence of septic shock on admission was the most strongly associated with risk of mortality among those factors. Unlike prognostic factors that cannot be changed, such as age, facility, and underlying diseases, outcomes might be improved with medical intervention for septic shock. Alcohol abuse is another preventable risk factor for pneumococcal disease, particularly among young adults^40^. This study also revealed alcohol abuse as a risk factor for mortality. Pneumococcal vaccination with PCV13 or PPV23 in adults is cost-effective and should be considered a priority for decision-makers^41^, and status of vaccination varied widely among studies (0–54.5%). Pneumonia vaccination was another effective means of preventing IPD, but much work remains to be done to increase the acceptance of pneumonia vaccination.

## Limitations

Several limitations to this study must be considered when interpreting the results. Given the nature of the disease in question, only a limited number of prospective studies were able to be enrolled. There posed a substantial risk for selection bias, due to the nature of observational study and risk factors analyzed in this manuscript were selected post-hoc. Second, the definition of mortality in articles as in-hospital or 30-day mortality was merged together in some risk factors, due to limited numbers of articles. Third, prognostic factors for mortality differed markedly between articles. Fourth, the forms of vaccine status for studies included in this meta-analysis varied. Fifth, the effects of serotype and antibiotic resistance were not discussed in the present investigation.

## Conclusion

IPD still shows high mortality rates and the presence of septic shock represents one of the most important prognostic factors for mortality in IPD. Unlike prognostic factors that cannot be changed, such as age, facility, and underlying diseases, early intervention for septic shock might improve the mortality in IPD.

## Supplementary Information

Supplementary Information.
