# Supplementary material for: Prognostic factors for mortality in invasive pneumococcal disease in adult: a system review and meta-analysis
Source: Sci Rep. 2021 Jun 4;11:11865. doi: 10.1038/s41598-021-91234-y (PMC8178309; doi:10.1038/s41598-021-91234-y)
Supplement: Supplementary file 1 — Supplementary Information. [file 41598_2021_91234_MOESM1_ESM.docx]

**Supplementary figure S1**

**Supplementary figure S2**

**Supplementary figure S3**

**Supplementary figure S4**
